# Drift versus selection as drivers of phenotypic divergence at small spatial scales: The case of Belgjarskógur threespine stickleback

**DOI:** 10.1002/ece3.5381

**Published:** 2019-06-27

**Authors:** Mathew Seymour, Katja Räsänen, Bjarni K. Kristjánsson

**Affiliations:** ^1^ Department of Aquaculture and Fish Biology Hólar University Skagafjörður Iceland; ^2^ Department of Aquatic Ecology EAWAG and Institute of Integrative Biology ETH‐Zurich Dübendorf Switzerland; ^3^ Molecular Ecology and Fisheries Genetics Laboratory School of Biological Sciences Bangor University Bangor UK

**Keywords:** adaptive divergence, FST, multivariate phenotype, plasticity, PST, stickleback

## Abstract

Divergence in phenotypic traits is facilitated by a combination of natural selection, phenotypic plasticity, gene flow, and genetic drift, whereby the role of drift is expected to be particularly important in small and isolated populations. Separating the components of phenotypic divergence is notoriously difficult, particularly for multivariate phenotypes. Here, we assessed phenotypic divergence of threespine stickleback (*Gasterosteus aculeatus*) across 19 semi‐interconnected ponds within a small geographic region (~7.5 km^2^) using comparisons of multivariate phenotypic divergence (PST), neutral genetic (FST), and environmental (EST) variation. We found phenotypic divergence across the ponds in a suite of functionally relevant phenotypic traits, including feeding, defense, and swimming traits, and body shape (geometric morphometric). Comparisons of PSTs with FSTs suggest that phenotypic divergence is predominantly driven by neutral processes or stabilizing selection, whereas phenotypic divergence in defensive traits is in accordance with divergent selection. Comparisons of population pairwise PSTs with ESTs suggest that phenotypic divergence in swimming traits is correlated with prey availability, whereas there were no clear associations between phenotypic divergence and environmental difference in the other phenotypic groups. Overall, our results suggest that phenotypic divergence of these small populations at small geographic scales is largely driven by neutral processes (gene flow, drift), although environmental determinants (natural selection or phenotypic plasticity) may play a role.

## 1 INTRODUCTION

Phenotypic variation of natural populations results from a combination of evolutionary processes, particularly gene flow, drift, natural selection, and environmentally induced phenotypic plasticity (Endler, 1986). Natural selection facilitates adaptation of populations in response to localized environmental or competitive pressure (Schluter, 2001) and by reducing gene flow (Räsänen & Hendry, 2008). In contrast, gene flow can counteract adaptive phenotypic divergence by homogenizing genotypic and phenotypic variation across environmental landscapes (Räsänen & Hendry, 2008; Slatkin, 1987). Less acknowledged is that the random loss of genetic diversity over generations (i.e., genetic drift) may also lead to phenotypic divergence (Lynch, 2007). While less noticeable across large heterogeneous landscapes, genetic drift may play an important role in early stage divergence, especially in small populations with limited gene flow (Wright, 1982).

Studies assessing adaptive divergence and the mechanisms of phenotypic divergence are most commonly conducted across large spatial (Belinda & Sgrò, 2017; Saether et al., 2007) and environmental (Belinda & Sgrò, 2017; Wilson, Peters, & McCracken, 2012) scales, where populations are expected to be under some degree of natural selection or dispersal limitation. Such large‐scale studies clearly indicate that natural selection across environmentally heterogeneous landscapes, often with nonrandom dispersal, facilitates phenotypic divergence, whereas local processes are often less considered (reviewed in Richardson, Urban, Bolnick, & Skelly, 2014). However, different evolutionary mechanisms do not act in isolation in natural populations, and the relative contribution of drift needs to be assessed to understand the sources of phenotypic differentiation (Clegg & Phillimore, 2010). Whereas the effects of natural selection and gene flow are more apparent in larger populations across heterogenous landscapes, drift is likely to be a particularly important mechanism in driving phenotypic divergence of small populations harboring low genetic diversity, concomitant to small spatial scales where environmental conditions are likely more homogenous (Hallatschek, Hersen, Ramanathan, & Nelson, 2007; Mayr, 1963; Nei & Tajima, 1981).

One way to assess the relative contribution of natural selection and neutral processes (i.e., gene flow and drift) on phenotypic variation, within and among natural populations, is to compare quantitative trait (QST) and neutral genetic (FST) variation (Leinonen, McCairns, O'Hara, & Merilä, 2013). If QST > FST, then divergent natural selection is likely driving phenotypic divergence. If QST = FST, genetic drift is assumed to play a primary role, whereas if QST < FST, stabilizing selection is believed to be at play (Brommer, 2011; Leinonen et al., 2013; Spitze, 1993). Moreover, comparing population pairwise QST and FST estimates in relation to population pairwise estimates of ecological variation (EST) allows insight into the relative roles of divergent natural selection and neutral processes (selection vs. gene flow vs. drift) influencing phenotypic variation within and among populations (Hangartner, Laurila, & Räsänen, 2012; Kaeuffer, Peichel, Bolnick, & Hendry, 2012). Estimating QSTs requires rigorous assessment of additive genetic variance (*c*) and narrow sense heritability (*h* ^2^), typically using quantitative genetic breeding designs, which are often not measurable for natural populations. PST, a phenotypic variance‐based measure of divergence, provides a field‐based proxy for QST (Brommer, 2011). Although not typically used in this context, PST estimates can also provide initial insights to the role of the environment (phenotypic plasticity and natural selection) on phenotypic variation when compared to ESTs (Kaeuffer et al., 2012).

While PST estimates are not as robust as QSTs, they do offer a means to assess phenotypic divergence of natural populations and have been applied in several studies (Kaeuffer et al., 2012; Leinonen, Cano, Makinen, & Merilä, 2006; Raeymaekers, Houdt, Larmuseau, Geldof, & Volckaert, 2007; Sæther et al., 2007). However, existing PST‐FST comparisons are typically based on univariate traits (meristic or traditional morphometric measurements), which do not account for the multivariate trait complexities that are involved in phenotypic expression, such as homeostasis or canalization (Forsman, 2014). Multivariate statistics are routinely used in ecology and molecular ecology to characterize total variation within and among populations, including geometric morphometrics (Mitteroecker & Gunz, 2009), genetic differentiation (Hartl & Clark, 1997), and species diversity (Seymour, Deiner, & Altermatt, 2016). Applying a multivariate PST‐FST comparison offers a more complete assessment of organism level phenotypic variation as opposed to comparing variation across several univariate phenotypic traits (Forsman, 2014; Spitze & Sadler, 1996).

**Figure 1:**
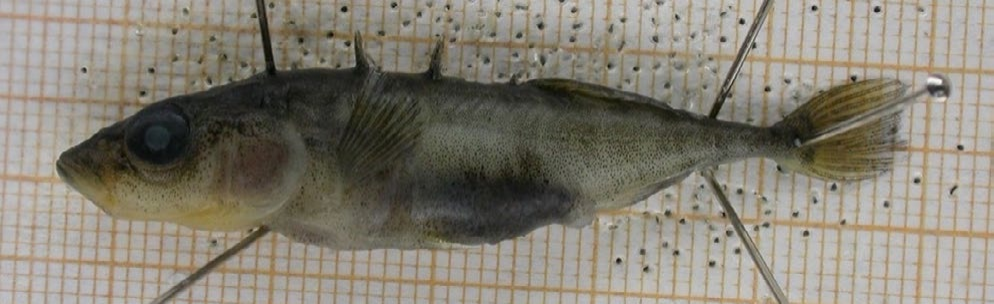
A representative threespine stickleback from a Belgjarskógur pond

**Figure 2:**
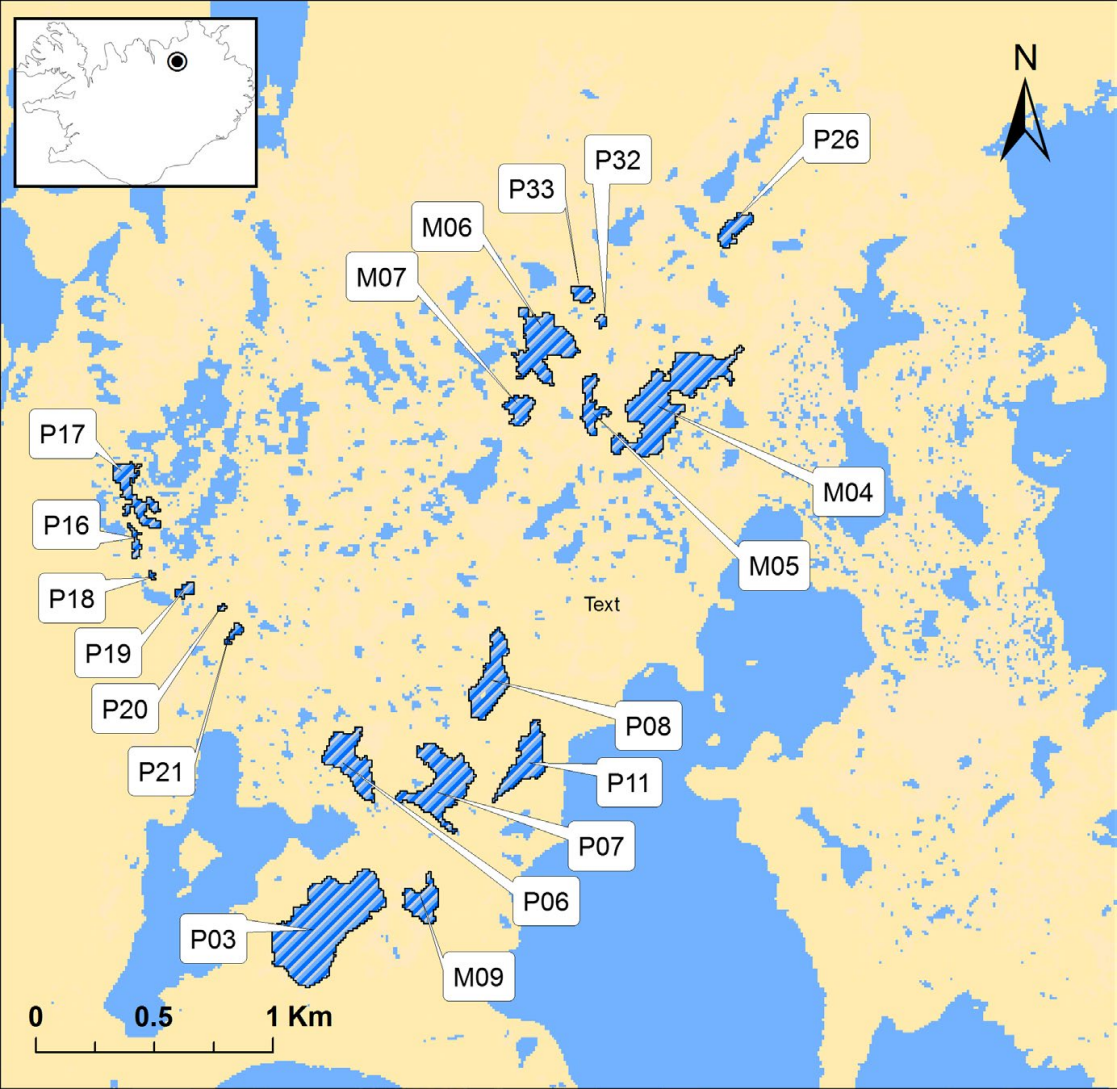
Map of the study area in Belgjarskógur, Iceland, showing 19 study ponds harboring threespine stickleback. The blue hatched areas indicate the study ponds. Top left insert shows an outline of Iceland and the geographic location of Belgjarskógur area represented as a black dot

Here, we implement PST‐FST and PST‐EST comparisons using a multivariate approach on threespine stickleback (*Gasterosteus aculeatus*) inhabiting a small geographic area in Iceland (Seymour, Räsänen, Holderegger, & Kristjánsson, 2013). Freshwater threespine stickleback (Figure 1) are well‐suited for such studies because they frequently occur in a range of water bodies that differ in connectivity, size, and environmental conditions. Moreover, phenotypic divergence of threespine stickleback across a range of morphological and life‐history traits can occur within only a few generations (Barrett et al., 2011; Bell, 1982; Kristjánsson, 2005). Icelandic threespine stickleback are diverse (Kristjánsson, Skulason, & Noakes, 2002; Lucek, Kristjánsson, Skúlason, & Seehausen, 2016; Ólafsdóttir, Snorrason, & Ritchie, 2007), partly due to the high diversity of Icelandic freshwater systems, caused by the interplay of glaciation and volcanic activity (Thorarinsson, 1979). We recently showed that neutral population genetic structure of threespine stickleback across a small pond system (Belgjarskógur, NE Iceland; Figure 2) is influenced by pond isolation and periodic connectedness, due to periods of flooding and drought facilitating or constraining gene flow (Seymour et al., 2013). Belgjarskógur was presumably formed within the last 2,300 years following a volcanic eruption in an area where lava fields—in close connection with groundwater—have created a complex wetland and pond landscape (Einarsson, 1982). Estimates of effective population size (Ne) further indicate that threespine stickleback populations in this area are generally small (Seymour et al., 2013, see below), making this system well‐suited to investigate the relative role of environmental selection/plasticity, gene flow, and drift in phenotypic divergence across small spatial scales.

Here, we assessed the relative contribution of environmental and neutral processes in threespine stickleback within the Belgjarskógur pond system. First, we measured the extent of phenotypic diversification of threespine stickleback across 19 study ponds, focusing on three multivariate meristic/morphometric trait groups (defense, feeding, and swimming traits) as well as body shape using geometric morphometric. Second, to infer whether selective or neutral processes influenced phenotypic divergence, we assessed the relationship between each set of phenotypic traits and neutral genetic divergence using PST‐FST comparisons. Third, we investigated the potential effects of environment (influencing both natural selection and phenotypic plasticity) by comparing PSTs to environmental differences (ESTs).

## 2 METHODS

### 2.1 Study system

Belgjarskógur, northeast of lake Mývatn, Iceland (Figure 2), is a geologically young wetland system (Thorarinsson, 1979) consisting of over a hundred lakes and ponds of various sizes (from a few square meters to ~105,000 m^2^) on a small geographic scale (~7.5 km^2^). The system was formed within the last 2,300 years, after lava from the Þrengslaborgir eruption flowed over a large part of the former Lake Mývatn (Einarsson, 1982). Most of the ponds in Belgjarskógur are inhabited by threespine stickleback (Figure 1), which are expected to have colonized the pond system from a single source, most likely from Lake Mývatn, or from the same ancestral source as the lake, followed by the division into many subpopulations.

Our previous study found variable genetic structuring among 19 of the ponds (pairwise FSTs across ponds ranged from 0.007 to 0.141), which was correlated with landscape connectivity associated with periodic flooding events (Figure 2; Seymour et al., 2013). Specifically, threespine stickleback in the western part of the system (Figure 2) showed stronger genetic structure, whereas threespine stickleback in the eastern part were more admixed. The studied ponds ranged from 608 to 105,000 m^2^ in size, and estimates of effective population size (*N*_e_) indicated small populations (*N*_e_ = 12–86; Seymour et al., 2013) and high potential for local genetic bottlenecks.

### 2.2 Sampling

Threespine stickleback (Figure 1) were collected from 19 ponds in June 2009 (Figure 2) using un‐baited minnow traps laid overnight. The collection process was repeated daily until at least 30 adult size (>30 mm in total length) threespine stickleback were caught in each study pond. Upon capture, threespine stickleback were euthanized using an overdose of phenoxyethanol, measured for total length (to the nearest 0.1 cm), and then frozen for later morphological and genetic analyses. After eliminating individuals due to physical deformations or destroyed feeding structures (due to freezing and storage), the final sample size was 15 to 47 individuals per pond (3–24 males and 7–30 females per pond, total *N* = 670). Removal of deformed animals was done to avoid errors in subsequent analyses that would not reflect natural population variation.

### 2.3 Environmental measurements

To characterize the environment that threespine stickleback experience, we recorded water chemistry parameters, pond size, and macroinvertebrate community diversity. Water chemistry was assessed for each pond using a YSI 600 XLM multiprobe sonde (YSI Incorporate) to measure pH (±0.2 units) and total dissolved solids (TDS; ppm). Chemistry was measured once in late June 2009 at each study pond. Total dissolved solids (TDS) were log_10_ transformed for statistical analyses (see below). To assess pond size, surface area of each pond was estimated from high‐resolution (2.5‐m pan‐sharpened spatial resolution) SPOT‐5 satellite imagery obtained from the National Land Survey of Iceland for the years 2002/2003 and ARCGIS 10 (ESRI, USA).

### 2.4 Invertebrates

To describe the feeding environment of threespine stickleback within each pond, invertebrate samples were collected just below the water surface and above the pond bottom by moving a hand net (153 micron mesh) for three minutes back and forth while moving forward at a slow pace. Samples were then rinsed and stored in 70% ethanol for later identification. All invertebrates retained in these samples were identified to various taxonomic levels (phylum: Mollusca, Nematoda; class: Acari, Coleopteran larvae, Collembola, Copepoda; order: Cladocera, Hymenoptera, Trichopteran larvae; family: Aphidoidea, Chironomidae larvae) using a Leica MZ12 (Nussloch) stereo microscope. If a given sample consisted of 200 or fewer individuals, all individuals were identified. If a given sample consisted of more than 200 individuals, the sample was thoroughly mixed and divided into aliquots. All individuals within an aliquot were then counted and the number of individuals in that aliquot multiplied by the total number of aliquots. If the number of individuals in the first aliquot was less than 200, the next aliquot was counted, and so on, until there were more than 200 individuals prior to calculating the total number. For statistical analyses, invertebrates were classified into three groups: limnetic (Copepoda, Cladocera), benthic (Mollusca, Nematoda, Acari, Coleopteran larvae, Chironomidae larvae, Trichoptern larvae), and “other” (Collembola, Hymenoptera, Aphidoidea) following (Schluter & McPhail, 1992).

### 2.5 Stomach content

To characterize diet, threespine stickleback stomachs were extracted from 670 individuals across the 19 study populations (10–30 individuals/pond). Stomachs were opened and contents scraped out with forceps to insure all contents were removed. All invertebrates retained in these samples were counted and identified to the lowest possible taxonomic level, using the same groups as above, using a Leica MZ12 stereo microscope. Partially digested items were not always identifiable and softer bodied prey (such as copepods or worms) were rarely found, whereas shells of Cladocera, Mollusca, and Chironomidae larvae, which take longer to digest, were more frequent. For statistical analyses, prey items were classified into limnetic, benthic, and other using the same groupings as for the pond invertebrate sampling.

### 2.6 Phenotypic variation

Threespine stickleback phenotypic variation was characterized by measuring linear morphometric traits or counts of meristic traits, as well as using geometric morphometrics to assess body shape. For phenotypic analyses, threespine stickleback were thawed, their stomachs removed, sexed by examining gonad morphology, and preserved in 70% ethanol, and subsequently fixed in 5% buffered formalin for three weeks. Prior to further processing, the formalin‐fixed fish were rinsed with water and transferred to 70% ethanol. For geometric morphometric analyses of shape, each formalin‐fixed fish was pinned onto a wax board, with spines spread out and mouth closed, and photographed on the left side using a Nikon (Tokyo, Japan) Coolpix 4500 digital camera (4 megapixels, Figure 1). Fish were subsequently bleached (1:1 ratio of 3% H_2_O_2_ and 1% KOH) and stained (Alizarin red 1% KOH) to aid measurement of traditional morphometric and meristic traits.

#### 2.6.1 Feeding morphology

For morphometric/meristic measurements of feeding traits (gill raker number, gill raker length, gill raker gap width), the first gill arch from each stained fish was removed, placed between two glass plates to assure a straight position, and photographed using a Nikon Coolpix 4500 (Nikon) mounted onto Leica MZ12 stereo microscope. Total gill raker number (GRN) across the long and short gill raker arcs, the length of the first five gill rakers on the long arc (GRL) and the width of the gaps between these five gill rakers (GW) were measured (to the nearest 0.1 mm) from the gill raker pictures using the public domain ImageJ software (Abramoff, Magelhaes, & Ram, 2004). Individual average GRLs and average GWs were then calculated for subsequent analyses.

#### 2.6.2 Swimming morphology

Swimming morphometric traits were assessed by measuring the length of the pectoral, anal, caudal, and dorsal fins (to the nearest 0.1 mm) using an ocular micrometer mounted on a Leica MZ12 stereo microscope and counting the number of fin rays on each fin.

#### 2.6.3 Defense morphology

Defense traits were characterized by counting the number of lateral plates from the left side of the fish and measuring the length of both long dorsal spines (to the nearest 0.1 mm) using an ocular micrometer. Average spine length was used in subsequent statistical analyses.

#### 2.6.4 Body shape

Body shape of each fish was assessed using geometric morphometrics using the program suite TPS (Rohlf, 2006). Before digitizing landmarks, photographs of the fish were randomly ordered and 11 fixed landmarks digitized on each photograph using the program TpsDig2. Landmarks were selected based on key morphological characteristics, focusing on the head (to capture trophic morphology) and fins (to capture locomotion morphology). Individuals were not included in these analyses if the mouth of the fish was open or the image was of poor quality, which would have reduced the accuracy of placing landmarks. This resulted in 18 fish being removed from the analysis (final *N* = 643). Digitized fish were aligned using generalized Procrustes superimposition using the geomorph package in R (Adams & Otárola‐Castillo, 2013). Briefly, a generalized Procrustes analysis (GPA) aligns all specimens based on position and angle and scales each specimen to a common size. The final Procrustes coordinates for each fish represents the size‐free shape of each fish (Rohlf & Bookstein, 2003). A PCA of the Procrustes coordinates was then calculated following Caumul and Polly (2005), which were used as the body shape matrix for the corresponding multivariate shape PST statistics detailed below.

### 2.7 Population genetic variation

Neutral genetic variation was assessed using 12 microsatellites (average allelic richness 2.7–4.04) to assess dispersal patterns in relation to landscape cover as previously reported (Seymour et al., 2013). These analyses found strong indications of isolation by distance, as determined through least cost pathways through interpolated floodplains. The average pond pairwise FSTs was 0.084 (Seymour et al., 2013), and we use here use pond pairwise FSTs from Seymour et al. (2013) as a measure of neutral genetic divergence.

### 2.8 Statistical analyses

All statistical analyses were performed in R (version 3.5.1, R Development Core Team, 2018). Morphometric measures that correlated with body size (i.e., GRL, GW, gill raker arc length, spine, and fin lengths) were size corrected following Reist (1986) by calculating size‐corrected residuals for each trait. Size‐corrected residuals were then used as response variables in further statistical analyses for these traits.

#### 2.8.1 Phenotypic divergence

We first assessed morphological variation among all ponds using multivariate analyses of variance (MANOVA). For each MANOVA, we assessed the effect of pond identity (19 levels) and sex (male and female) on the response trait matrix (feeding, swimming, and defense) using the *lm* function in R. Because of the small sample size for males or females in some populations, we did not test for the population and sex interactions. Subsequently, we assessed individual trait variation among populations using ANOVAs, which are a special case of linear regression, via the ANOVA function in R, with pond identity and sex as explanatory variables. Response variables included body length, pectoral fin length, dorsal fin length, caudal fin ray number, pectoral fin ray number, GRN, GRL, GW, dorsal spine length, and number of lateral plates. Length measurements were size corrected as previously mentioned. Geometric morphometric shape variation across the ponds was assessed using Procrustes ANOVAs with the procD.lm function in the geomorph package, with Procrustes coordinates as response variables and pond identity and sex as the explanatory variables, with 1,000 residual randomized permutations. Procrustes ANOVA allows for statistical assessment of the term (here: pond identity and sex) using Procrustes distances among specimens and is equivalent to distance‐based ANOVA, allowing comparison with the analyses of the morphometric/meristic variables stated prior (Anderson, 2001). To check for allometric trends across the ponds, we performed a preliminary Procrustes ANOVA that included centroid size × pond identity interaction as a covariate. While we found a significant difference in centroid size across ponds (*p* < 0.01) and pond identity (*p* < 0.01), we also found a significant effect of centroid × pond identity interaction (*p* = 0.036), indicating that allometric relationships are not the same in all the ponds. Therefore, we elected to not to include size as a co‐variant for the final analysis due to the nongeneral allometric trends across the ponds and underlying importance of the allometric variation in describing the observed phenotypic variation (Klingenberg, 2016).

#### 2.8.2 PST calculation

**Figure 3:**
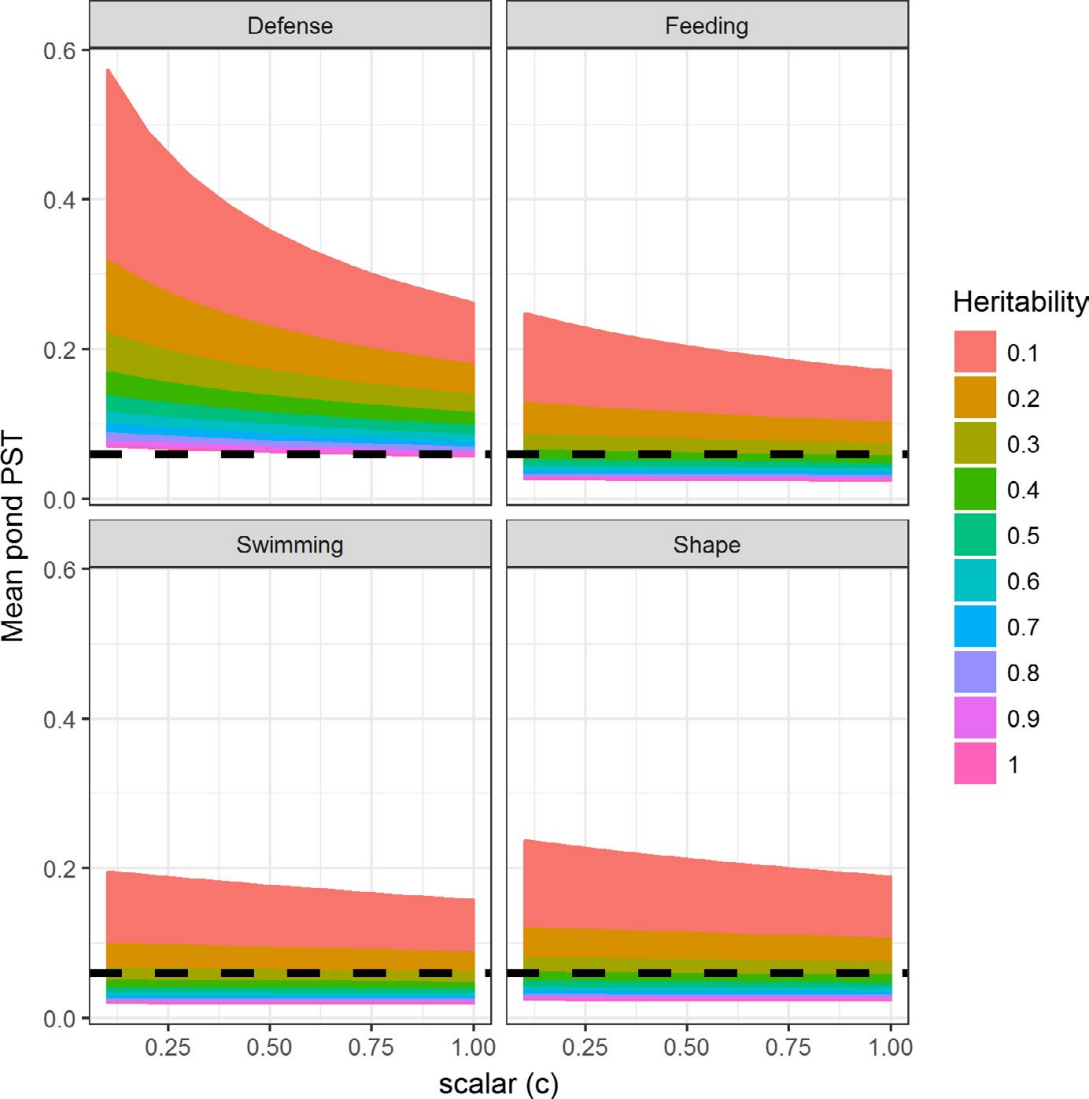
Sensitivity of the mean PST estimates, relative to mean FST (black dotted line). Estimated PSTs ranges (colored lines) are represented for the 19 ponds for each set of phenotypic traits (panels), assuming different trait heritabilities (*h* ^2^, legend) and additive genetic values (scalar = *c*, *x*‐axes). If mean PST > FST, then natural selection (or phenotypic plasticity, if wild collected individuals) is likely driving phenotypic divergence. The figure shows that PST > FST for most *h* ^2^ values for defense traits and for *h* ^2^ < 0.4 for feeding, swimming, and shape traits. Moreover, at low *h* ^2^, PST estimates are more strongly dependent on assumed additive genetic variance (c), especially for defense phenotype

PST was calculated for each pond using the formula:

$$\text{PST}=\delta_{\text{GB}}^{2}/\left(c*\delta_{\text{GB}}^{2}+2*h^{2}*\delta_{\text{GW}}^{2}\right),$$

where $\delta_{\text{GB}}^{2}$ is the variation between ponds, $\delta_{\text{GW}}^{2}$ is the variation within ponds, *h* ^2^ is trait heritability, and *c* is a unit‐less scalar variable (Brommer, 2011). Variation in traits between and within each pond pair were calculated using redundancy analysis (RDA), which partitioned the within and among pond variation of each multivariate trait matrix (the response variable) with pond as the among group explanatory variable. We used heritability of 0.5 (i.e., 50% of phenotypic variation is genetically based and due to additive genetic variance) and a scalar value of 1 (i.e., 100% of variance among populations is due to additive genetic variance, Brommer, 2011) for further analyses. These assumed values are commonly used in other QST and PST studies (Brommer, 2011; e.g., Leinonen et al., 2013). However, we examined the change in PST across all combinations of a range of heritabilities and scalar values (from 0.1 to 1 in 0.1 increments). This showed us that changing these variables did not greatly influence the results (Figure 3). Furthermore, heritability estimates of different traits in threespine stickleback vary from relatively low (plate count: *h* ^2^ = 0.25, (Mc Cairns & Bernatchez, 2012) and *h* ^2 ^= 0.26, (Baumgartner, 1995; Leinonen, Cano, & Merilä, 2011)) to intermediate (fin length: *h* ^2 ^= 0.46 (Loehr, Leinonen, Herczeg, O'Hara, & Merilä, 2012), gill raker number: *h* ^2^ = 0.58, (Leinonen et al., 2011) spine length: *h* ^2 ^= 0.61 to 0.66 (Leinonen et al., 2011; Loehr et al., 2012)). Very high heritabilities are rare, but *h* ^2^ = 0.84 has been recorded for plate counts in some populations (Hagen, 1973).

#### 2.8.3 PST versus FST

We first calculated the mean PST and FST for each pond from the initial pairwise PST or FST calculations (see above) to avoid violating the assumption of independence in the subsequent linear models. These pond mean PSTs and FSTs were then compared, using the lm function in R to perform a linear regression, with FST as the response variable and PST as the explanatory variable. Given that our study utilizes a set of sampling sites that is large enough to compare means across all populations, we opted for a mean‐based comparison of PST‐FST and PST‐EST. Assessing population means is a conservative approach compared to using analyses of pairwise distance matrices, such as Mantel test (Guillot & Rousset, 2013). We tested the statistical difference between pond mean PSTs and FST using the ANOVA function in R, with pond mean values (*N* = 19) as the response and FST/PST group as the explanatory. We removed two outlier points (final *N* = 17) for the defense PST versus FST significant test to maintain the assumption of normality for the ANOVA and to ensure the outliers were not influencing the test.

#### 2.8.4 PST‐EST

Environmental divergence (EST) here refers to observed ecological gradients (e.g., Kaeuffer et al., 2012) in (a) pond invertebrate community, (b) threespine stickleback diet, and (c) abiotic parameters. For the invertebrate and dietary analyses, mean proportion of limnetic prey to benthic prey was calculated for each pond. The abiotic gradient was calculated as the first principal component of a PCA that included pH, total dissolved solids, and pond surface area. Total dissolved solids and pond surface area were normalized, using min–max scaling, prior to the PCA calculation (pH was normally distributed). Subsequently, for each set of pond mean PSTs (for each trait type) and pond ESTs (for each environmental parameter), a linear regression was performed to assess the effect of EST on PST using the lm function in R, as previously stated for the PST‐FST analysis.

## 3 RESULTS

### 3.1 Environmental variation

Total dissolved solids ranged from 0.042 to 0.306 ppm, pH from 7.26 to 9.38 (reflecting neutral to alkaline ponds), and pond surface area from 608 to 105,000 m^2^ across the ponds. Proportions of sampled limnetic to benthic invertebrates were high across the ponds (mean proportion per pond = 0.887; *SD* = 0.142) The dietary analysis showed lower proportions of limnetic to benthic prey across ponds, with mean proportion of limnetic per pond being 0.43; *SD* = 0.21.

### 3.2 Morphological variation

Average body size of threespine stickleback varied between males (mean = 47.9 mm, *SD* = 6.4) and females (mean = 51.9 mm, *SD* = 8.0) and ranged from 44 to 57 mm across the ponds (mean = 50.3 mm *SD* = 3.6).

#### 3.2.1 Feeding traits

Multivariate feeding traits varied significantly among ponds (Pillai = 0.703, *p* < 0.01) and sex (Pillai = 0.057, *p* < 0.01). Population mean GRN ranged from 12.3 to 14 (mean = 13.1, *SD* = 0.35), size‐corrected GRL from 0.76 to 1.02 mm (mean = 0.89, *SD* = 0.10), size‐corrected GW from 0.18 to 0.22 mm (mean = 0.19, *SD* = 0.01), and size‐corrected arc length from 1.40 to 1.72 mm (mean = 1.56, *SD* = 0.10). GRN, GRL, and arc length differed also between sexes (all *p* < 0.05), whereas GW did not (*p* = 0.07; Table 1).

**Table 1:** Analyses of variance (ANOVA) for effects of pond identity on meristic phenotypic traits, grouped by trait type (body length, swimming, feeding, and defense) and Procrustes ANOVA of the effects of pond identity on the geometric morphometric‐derived Procrustes coordinate matrix of body shape

		Sum Sq	Mean Sq	*F*	*p*
*Length*	Pond	2.987	0.166	9.53	**<0.01**
	Sex	0.822	0.822	47.23	**<0.01**
*Swimming*					
PFL	Pond	17.217	0.957	4.79	**<0.01**
	Sex	8.607	8.607	43.06	**<0.01**
DFRN	Pond	0.447	0.025	2.78	**<0.01**
	Sex	0.109	0.109	12.16	**<0.01**
TFRN	Pond	0.287	0.016	2.03	**<0.01**
	Sex	0.04	0.04	5.13	**0.02**
PFRN	Pond	0.012	0.001	0.73	0.78
	Sex	0.003	0.003	3.31	0.07
*Feeding*					
GRN	Pond	1.581	0.088	3.39	**<0.01**
	Sex	0.143	0.143	5.52	**0.02**
GRL	Pond	3.991	0.222	17.06	**<0.01**
	Sex	0.42	0.42	32.29	**<0.01**
GL	Pond	0.051	0.003	3.61	**<0.01**
	Sex	0.003	0.003	3.26	0.07
AL	Pond	6.379	0.354	9.84	**<0.01**
	Sex	0.217	0.217	6.02	**0.01**
*Defense*					
DSL	Pond	11.791	0.655	7.2	**<0.01**
	Sex	1.55	1.55	17.05	**<0.01**
LPN	Pond	25.113	1.395	8.8	**<0.01**
	Sex	1.154	1.154	7.28	**<0.01**
*Shape*	Pond	0.115	0.006	3.75	**<0.01**
	Sex	0.002	0.002	1.31	0.16

Note

Pond DF = 18, Sex DF = 1, Residuals = 642. Statistically significant effects (*p* < 0.05) are highlighted in bold.

Trait abbreviations: AL, arc length; DFRN, dorsal fin ray number; DSL, dorsal spine length; GRL, gill raker length; GRN, gill raker number; GW, gill raker gap width; LPN, lateral plate number; PFL, pectoral fin length; PFRN, pectoral fin ray number; TFRN, tail fin ray number.

#### 3.2.2 Swimming traits

Multivariate swimming traits varied significantly among ponds (Pillai = 0.264, *p* < 0.01) and sex (Pillai = 0.085, *p* < 0.01). Size‐corrected pond mean pectoral fin length ranged from 6.00 to 6.53 mm (mean = 6.22, *SD* = 0.17), average dorsal fin ray numbers ranged from 11.1 to 11.8 (mean = 11.3, *SD* = 0.18), and average caudal fin ray numbers ranged from 11.9 to 12.5 (mean = 12.2, *SD* = 0.15). Pectoral fin length, dorsal fin ray number, and caudal tail fin ray number differed among populations (all *p* < 0.01, Table 1), while pectoral fin ray numbers did not (*p* = 0.78) (Table 1). Pectoral fin length, dorsal fin ray numbers, and caudal fin ray numbers (all *p* < 0.02, Table 1) differed between sexes, while pectoral fin ray number did not (*p* = 0.07).

#### 3.2.3 Defense traits

Multivariate defense traits varied significantly among ponds (Pillai = 0.358, *p* < 0.01) and sex (Pillai = 0.036, *p* < 0.01). Size‐corrected pond mean spine length ranged from 2.82 to 3.33 mm (mean = 3.1, *SD* = 0.15) and lateral plate number from 0.1 to 3.11 (mean = 0.97, *SD* = 0.74). Spine length as well as lateral plate number differed significantly among ponds (both *p* < 0.01) and sex (both *p* < 0.01, Table 1).

#### 3.2.4 Geometric morphometric body shape

**Figure 4:**
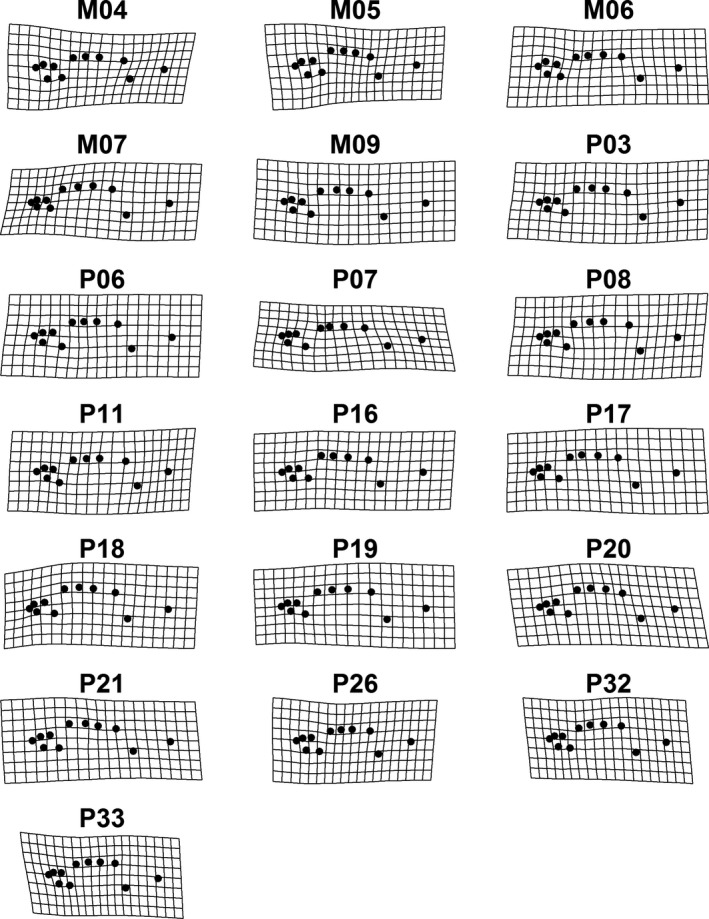
Geometric morphometric‐based Procrustes plots of the mean fish shape from each pond (M04 to P33) relative to the mean reference fish shape across all populations. Black dots correspond to landmarks

Procrustes ANOVA of the Procrustes coordinate matrix indicated threespine stickleback differed in body shape among ponds (*p* = 0.01, Table 1; Figure 4), but not between the sexes (*p* = 0.16).

### 3.3 PST‐FST and PST‐EST comparisons

We tested the effect of changing values for *h* ^2^ and *c* (Figure 3). The results were quite robust, justifying our selection of *h* ^2^ = 0.5 and *c* = 1. Mean PSTs across ponds for feeding morphology, swimming morphology, and body shape were greater than pond FSTs only when *h* ^2^ was low (*h* ^2^ < 0.2–0.3; Figure 3). In contrast, defense traits showed consistently greater mean PSTs than FSTs across the range of *h* ^2^ and *c* (Figure 3). Using a *h* ^2^ = 1 (i.e., trait variation among populations is fully genetically determined) generally greatly altered the magnitude and variation of the PST calculations, whereas altering *c* at *h* ^2^ < 1 had little effect on any of the PST calculations (Figure 3).

**Figure 5:**
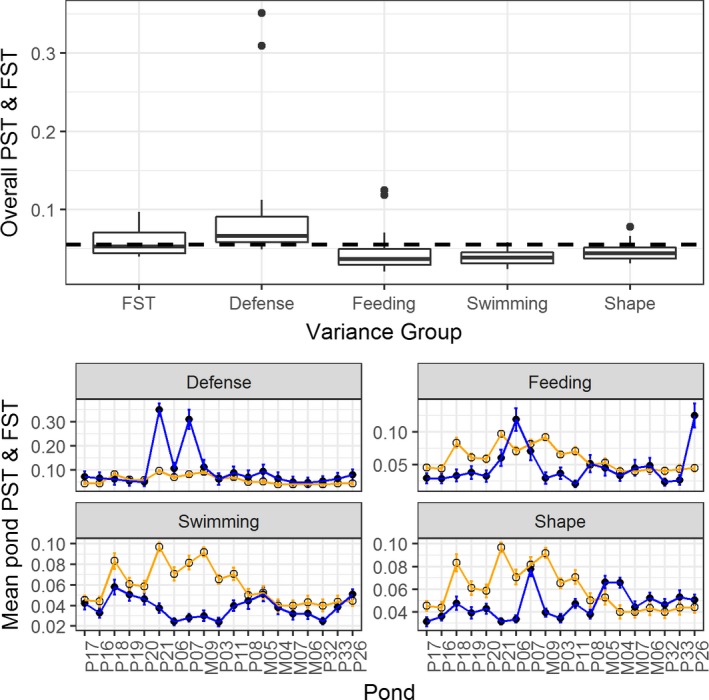
*Upper graph*: Box plots showing mean PST and FST values (*y*‐axis) across all 19 sampling ponds for each PST and FST groups (*x*‐axis). Dotted line is mean FST across all ponds to allow direct comparison between mean PST and FST values. The upper and lower whiskers correspond to the 1.5 times interquartile range. *Lower graphs*: pond‐specific PSTs and FSTs for each phenotypic trait type (defense, feeding, swimming, and body shape). The corresponding pond‐specific PSTs (blue line, closed points) and FSTs (orange line, open points) values (*y*‐axis) plotted for each pond (*x*‐axis). Error bars show the standard error for mean PSTs and FSTs for each pond calculated from all possible pairwise comparisons. Ponds are ordered geographically from west to east (*x*‐axis, Figure 2)

Pond‐specific PSTs for feeding morphology ranged from <0.001 to 0.181 (mean = 0.025, *SD* = 0.030), for swimming morphology from <0.001 to 0.060 (mean = 0.017, *SD* = 0.013), for defense morphology from <0.001 to 0.056 (mean = 0.057, *SD* = 0.086), and for body shape from 0.007 to 0.091 (mean = 0.026, *SD* = 0.014). Pond‐specific FSTs ranged from 0.007 to 0.141 (mean = 0.059, *SD* = 0.030; Figure 5).

Using *h* ^2^ = 0.5 in our calculation of PST, we found a significant positive relationship between defense PST and FST (*R* ^2^ = 0.404, *p* = 0.002), with defense PST significantly greater compared to FST (*p* = 0.02; Figure 5). We checked that the PST‐FST linear relationship in defense traits was not driven by outliers, by removing the two extreme populations and reanalyzing the model, whereby the results were still significant (*R* ^2^ = 0.245, *p* = 0.025). We found nonsignificant PST‐FST relationships for feeding (*R* ^2^ = −0.048,* p* = 0.687), swimming (*R* ^2^ = −0.058, *p* = 0.91), and shape (*R* ^2^ = −0.041, *p* = 0.596) traits (Table 2). Pond mean PSTs were significantly lower than FST for swimming traits (*p* < 0.01) and body shape (*p* = 0.02; Figure 5). Feeding traits pond mean PSTs were not significantly different from FST (*p* = 0.14; Figure 5).

**Table 2:** Linear regression statistics for effects of FST on PST, grouped by PST measure

PST‐FST					
Group	Sum Sq	Resid. Mean sq	*F* value	*R* ^2^	*p*
Defense	5.60E−02	7.18E−02	13.20	0.404	0.002
Feeding	1.51E−04	1.52E−02	0.17	−0.048	0.687
Swimming	1.44E−06	1.84E−03	0.01	−0.058	0.91
Shape	4.91E−05	2.86E−03	0.29	−0.041	0.596

Note

Residual degrees of freedom = 17.

There was a positive relationship between PSTs of swimming morphology and ESTs represented as pond limnetic invertebrate communities (*R* ^2^ = 0.230, *p* = 0.022), and a positive relationship between PSTs and EST as limnetic prey (*R* ^2^ = 0.161, *p* = 0.050; (Table 3). All other PST‐EST relationships, including associations with abiotic variation, were nonsignificant (Table 3).

**Table 3:** Linear regression statistics for effects of EST on PST, grouped by PST measure, for (a) diet EST, (b) pond invertebrate EST, and (c) abiotic environment

Group	Sum Sq	Resid. Mean sq	*F* value	*R* ^2^	*p*
(a) PST‐EST (diet)					
Defense	2.61E−03	1.25E−01	0.355	−0.037	0.559
Feeding	2.51E−04	1.51E−02	0.282	−0.042	0.603
Swimming	3.83E−04	1.46E−03	4.456	0.161	0.050
Shape	6.64E−05	2.85E−03	0.397	−0.035	0.537
(b) PST‐EST (biotic)					
Defense	2.75E−03	1.25E−01	0.375	−0.036	0.549
Feeding	1.76E−04	1.52E−02	0.197	−0.047	0.663
Swimming	5.03E−04	1.34E−03	6.383	0.230	0.022
Shape	8.62E−06	2.90E−03	0.051	−0.056	0.825
(c) PST‐EST (abiotic)					
Defense	3.44E−03	1.25E−01	0.471	−0.037	0.502
Feeding	1.27E−03	1.48E−02	1.532	−0.019	0.233
Swimming	2.90E−04	1.57E−03	3.172	0.097	0.093
Shape	4.84E−04	2.52E−03	3.384	0.085	0.083

Note

Residual degrees of freedom = 17.

## 4 DISCUSSION

We found clear differences among phenotypes of threespine stickleback across 19 small, intermittently connected, ponds. These phenotypic differences were seen in meristic/morphometric feeding, defense, and swimming traits and in body shape (geometric morphometric). Phenotypic divergence (population pairwise PSTs) in defense traits was greater than neutral genetic differentiation (population pairwise FSTs), indicating divergent selection or phenotypic plasticity. There were no clear difference between phenotypic divergence and neutral genetic differentiation for swimming traits, feeding traits, or body shape, suggesting that divergence in these traits may be mostly influenced by genetic drift or stabilizing selection. We found little evidence for the role of environmental divergence (EST) in phenotypic divergence, which may partly reflect relative environmental similarities of the ponds across the study area. The exception was that divergence in swimming traits positively correlated with pond differences in the proportion of limnetic invertebrates in the environment and in stickleback diet. Overall, our results suggest that phenotypic divergence in these small populations is driven by a combination of neutral processes (gene flow, drift) and either natural selection or phenotypic plasticity in response to small‐scale environmental variation.

Defensive traits showed stronger phenotypic divergence relative to neutral genetic divergence, regardless of assumed heritability, which suggests that divergent selection or phenotypic plasticity may have promoted differentiation in defensive traits across our study ponds. The among pond divergence in these traits was most apparent among the southern and westernmost ponds, which have previously been shown to have greater dispersal limitation compared to the more admixed northeastern ponds (Seymour et al., 2013). In general, defensive traits (pelvic girdle, lateral plate number and size, spine lengths) in threespine stickleback are commonly under divergent selection via predation pressure (Barrett, Rogers, & Schluter, 2008). The defensive traits of ancestral marine threespine stickleback likely evolved when gape‐limited predators, including fish and birds, promoted the selection of long spines and numerous lateral plates (Bell & Foster, 1994). In freshwater populations, this selection has commonly been reversed, although there is considerable variation (Bell & Foster, 1994). In Belgjarskógur the predation of threespine stickleback is suspected to be primarily avian, for example, by the horned grebe (*Podiceps auritus*), red‐breasted merganser (*Mergus serrator*), and great northern diver (*Gavia immer*), which migrate to the area for breeding. Fish predation is expected to be rare in these ponds due to their small size. Larger ponds may contain Arctic charr (*Salvelinus alpinus*) and brown trout (*Salmo trutta*; Einarsson et al., 2004), who are possible predators on threespine stickleback. Small ponds may have greater vegetation cover, where threespine stickleback could more easily hide from possible predators, both avian and fish, in comparison with larger ponds. Lower plate numbers, which would indicate low predation, were observed in small western ponds; however, other fish in ponds of similar size also had plate numbers comparable to larger ponds.

Interestingly, we found that phenotypic divergence in swimming traits, feeding traits, and body shape was either promoted by drift (PST = FST) or under divergent selection (PST > FST). Commonly for QST‐FST comparisons, trait heritability is assumed to be 0.5 (e.g., Hangartner et al., 2012; Kaeuffer et al., 2012), although Brommer (2011) indicated that heritability is an important assumption that should be considered. All PST/QST analyses depend strongly on the assumed value for narrow sense heritability; however, studies commonly do not conduct sensitivity analysis for PST calculations. Here, we decided to include a sensitivity analysis of the PST calculation, which supported our selection of values for *h* ^2^ and *c*, but also highlights the importance of narrow sense heritability when calculating PST.

In threespine stickleback, highly heritable traits include lateral plate number, gill raker number, and dorsal fin rays, whereas body size, body shape, gill raker length, and gap width, as well as spine length, tend to be more plastic with narrow sense heritability ranging between 0.3 and 0.6 (Hermida, Fernandez, Amaro, & San Miguel, 2002). For this study, we assumed narrow sense heritability across the multivariate traits groups to be 0.5, based on existing literature and comparable PST/QST study assumptions. While assuming a mean narrow sense heritability of 0.5, which is comparable to heritability estimates in plastic length‐based threespine stickleback traits (Leinonen et al., 2011), it is likely that the more plastic phenotypes (lower heritability) in our study (i.e., body shape and swimming structures), are highly influenced by drift or potentially stabilizing selection (i.e., PST < FST). Traits that are likely less plastic (higher heritability), for example, feeding structures, are more likely influenced by drift and could possibly be under some stabilizing selection if we assumed an elevated *h* ^2^ of 0.6 or greater, although elevated heritability values above 0.5 are not common for these traits (Leinonen et al., 2011; Loehr et al., 2012).

### 4.1 Drivers of phenotypic divergence: environment versus neutral processes

We found that variation in swimming trait of stickleback was positively correlated with the relative abundance of limnetic prey (e.g., copepods and cladocerans) in the diet and the availability of limnetic prey in the ponds. Threespine stickleback morphology is often linked to availability of limnetic versus benthic prey (Bell & Foster, 1994), whereby stickleback develop longer gill rakers, larger fins (relative to body size), and more streamlined bodies to capture limnetic prey (Willacker, Hippel, Wilton, & Walton, 2010). While previous studies have shown clear benthic–limnetic divergence in threespine stickleback in several lakes across the northern hemisphere, the differentiation is much weaker within the shallow Lake Mývatn, where divergence of stickleback is related to benthic habitats (Millet, Kristjánsson, Einarsson, & Räsänen, 2013). Likewise, Belgjarskógur threespine stickleback swimming morphology may be a plastic response to more limnetic feeding strategies in order to chase limnetic prey that are more abundant in the water column (Day & McPhail, 1996)—as reflected in the similar relationship between swimming PST and prey availability and diet ESTs. Based on the PST‐FST and PST‐EST comparisons, swimming traits may be under stabilizing selection due combinations of small effective population size and seasonal changes in prey availability. Whereas, body shape and feeding traits seem to reflect drift (PST = FST) across small geographic and environmental scales (Whitney, Bowen, & Karl, 2018). Given the limited number of generations since Belgjarskógur separated from the larger Mývatn system (~2,500 threespine stickleback generations), the observed phenotypic variation between ponds may be a recent example of genetic drift resulting from genetic sorting and range expansion following localized extinction (Hallatschek et al., 2007).

## 5 CONCLUSIONS

Here, we examined the phenotypic divergence of threespine stickleback, across small populations under partially dispersal limited conditions and over a small geographic scales. Specifically, we examined whether different evolutionary processes may account for the observed variation in different sets of traits. Our findings show that genetic drift is likely a key player for a large extent of the observed phenotypic diversification. At the same time, environmental differences were correlated with small spatial scale phenotypic divergence in swimming morphology, although PST‐FST comparisons indicated that these traits were under stabilizing selection. Due to the small effective population sizes (*N*_e_ = 12–86, Seymour et al., 2013) and variation in connectivity of ponds across Belgjarskógur (Seymour et al., 2013), combinations of random genetic drift and local natural selection at small geographic scales in isolated populations are likely facilitating phenotypic divergence in this system. Overall, our findings highlight the importance of assessing variation in phenotypic and genetic variation on dispersal limited and geographically close systems to further our understanding of the relative roles of drift, gene flow, and natural selection in biological diversification.

## CONFLICT OF INTERESTS

Authors declare no competing interest.

## AUTHOR CONTRIBUTIONS

BKK, KR, and MS designed the study and wrote the paper. MS conducted the fieldwork, performed the laboratory and data analyses, and lead the writing. All authors commented on manuscript drafts.

## Data Availability

Data for the manuscript are available on dryad https://doi.org/10.5061/dryad.5vf368h.
